# CD46 protects the bladder cancer cells from cetuximab-mediated cytotoxicity

**DOI:** 10.1038/s41598-022-27107-9

**Published:** 2022-12-27

**Authors:** Manh-Hung Do, Hien Duong Thanh, Phuong Kim To, Min Soo Kim, Changjong Moon, Chaeyong Jung

**Affiliations:** 1 Department of Anatomy, Chonnam National University Medical School, Gwangju, 61469 Korea; 2 Department of Statistics, College of Natural Sciences, Chonnam National University, Gwangju, 61186 Korea; 3 College of Veterinary Medicine, Chonnam National University, Gwangju, 61186 Korea

**Keywords:** Targeted therapies, Bladder cancer

## Abstract

Epidermal growth factor receptor (EGFR) is an effective target for those patients with metastatic colorectal cancers that retain the wild-type *RAS* gene. However, its efficacy in many cancers, including bladder cancer, is unclear. Here, we studied the in vitro effects of cetuximab monoclonal antibodies (mAbs) targeting EGFR on the bladder cancer cells and role of CD46. Cetuximab was found to inhibit the growth of both colon and bladder cancer cell lines. Furthermore, cetuximab treatment inhibited AKT and ERK phosphorylation in the bladder cancer cells and reduced the expression of CD46 membrane-bound proteins. Restoration of CD46 expression protected the bladder cancer cells from cetuximab-mediated inhibition of AKT and ERK phosphorylation. We hypothesized that CD46 provides protection to the bladder cancer cells against mAb therapies. Bladder cancer cells were also susceptible to cetuximab-mediated immunologic anti-tumor effects. Further, cetuximab enhanced the cell killing by activating both antibody-dependent cellular cytotoxicity (ADCC) and complement-dependent cytotoxicity (CDC) in bladder cancer cells. Restoration of CD46 expression protected the cells from both CDC and ADCC induced by cetuximab. Together, CD46 exhibited a cancer-protective effect against both direct (by involvement of PBMC or complement) and indirect cytotoxic activity by cetuximab in bladder cancer cells. Considering its clinical importance, CD46 could be an important link in the action mechanism of ADCC and CDC intercommunication and may be used for the development of novel therapeutic strategies.

## Introduction

Epidermal growth factor receptor (EGFR) is a family of transmembrane receptor tyrosine kinases, which includes EGFR (HER1), HER2, HER3, and HER4. Ligand-induced homo- or heterodimerization of the receptors results in the cross-phosphorylation of dimers and triggers intracellular signaling of the RAS-RAF-MEK-ERK and PI3K-AKT axes^1–3^. Such signals play important roles in normal cell proliferation, differentiation, and apoptosis and cell invasion^4–6^. However, deregulation of EGFR signaling through mutations, overexpression, or gene amplification commonly results in development and progression of several human cancers and resistance to therapies^7,8^. Cetuximab is an IgG1 monoclonal antibody (mAb) that targets the extracellular domain of EGFR to block ligand binding and subsequently inhibit intracellular signaling^7^. Cetuximab ultimately induces cell death via two mechanisms—complement-dependent cytotoxicity (CDC) and antibody-dependent cellular cytotoxicity (ADCC)^9,10^. ADCC is the major mechanism of the mAbs—the Fab fragment of the mAbs binds to specific antigens on the surface of the target cells. The Fc fragment of the same mAb then binds to the CD16 Fc receptors on the effector cells (usually the natural killer (NK) cells), where degranulation of cytokines and apoptotic agents leads to the destruction of target cells. CDC functions in a similar manner, wherein mAbs bind to the antigens on the target cells and activate the complement cascade rather than recruiting NK cells. Activation of multiple complements induces the formation of membrane attack complex (MAC), resulting in cell lysis.

CD46 is a membrane-bound complement regulatory protein (mCRP) that protects the cells from non-specific CDC. The mCRPs include the membrane cofactor protein CD46, the decay-accelerating factor CD55, and the protectin CD59. The two important activation fragments of the complement system are C3b and C4b; the deposition of these proteins on the cell membrane is regulated by CD46. Further, CD46 acts as a cofactor for the serine protease factor I-mediated destruction of these two components and prevents their contribution in complement activation^11,12^. CD46 is highly overexpressed in various solid cancers of the stomach, ovary, breast, and bladder^13–18^. However, the role of CD46 in these cancers is not clearly understood. Complement products can contribute to ADCC by interacting with various complement receptors expressed on the immune effector cells. This indicates that all mCRPs directly block ADCC^19–22^.

Bladder cancer ranks tenth in terms of the highest morbidity rate worldwide^23^. In the United States, it is much more common, with the rankings of sixth and fourth in both sexes and men, respectively^23–25^. Its high rates of morbidity and recurrence, coupled with poor survival, make bladder cancer a serious public health concern. Conventional treatments for bladder cancer, such as chemotherapy and radiotherapy, have poor efficacy and unacceptable side effects. Therefore, novel, effective therapeutics with targeted and personalized approaches are necessary. Cetuximab is approved by the FDA for patients with KRAS wild-type metastatic colorectal cancer expressing EGFR and head and neck squamous cell carcinoma. Studies have revealed that the expression of EGFR varies widely from 26 to 74% in bladder cancer^26–28^. This overexpression contributes to disease progression and poor clinical outcomes^29,30^. The results of the current study supported the hypothesis that mAbs stimulate both CDC and ADCC via downregulating the complement inhibitory membrane cofactor protein CD46. Furthermore, it was found that bladder cancer cells treated with cetuximab inhibited CD46 expression and subsequently enhanced both CDC and ADCC.

## Results

### Bladder cancer cells are responsive to cetuximab-mediated cytotoxicity

**Figure 1:**
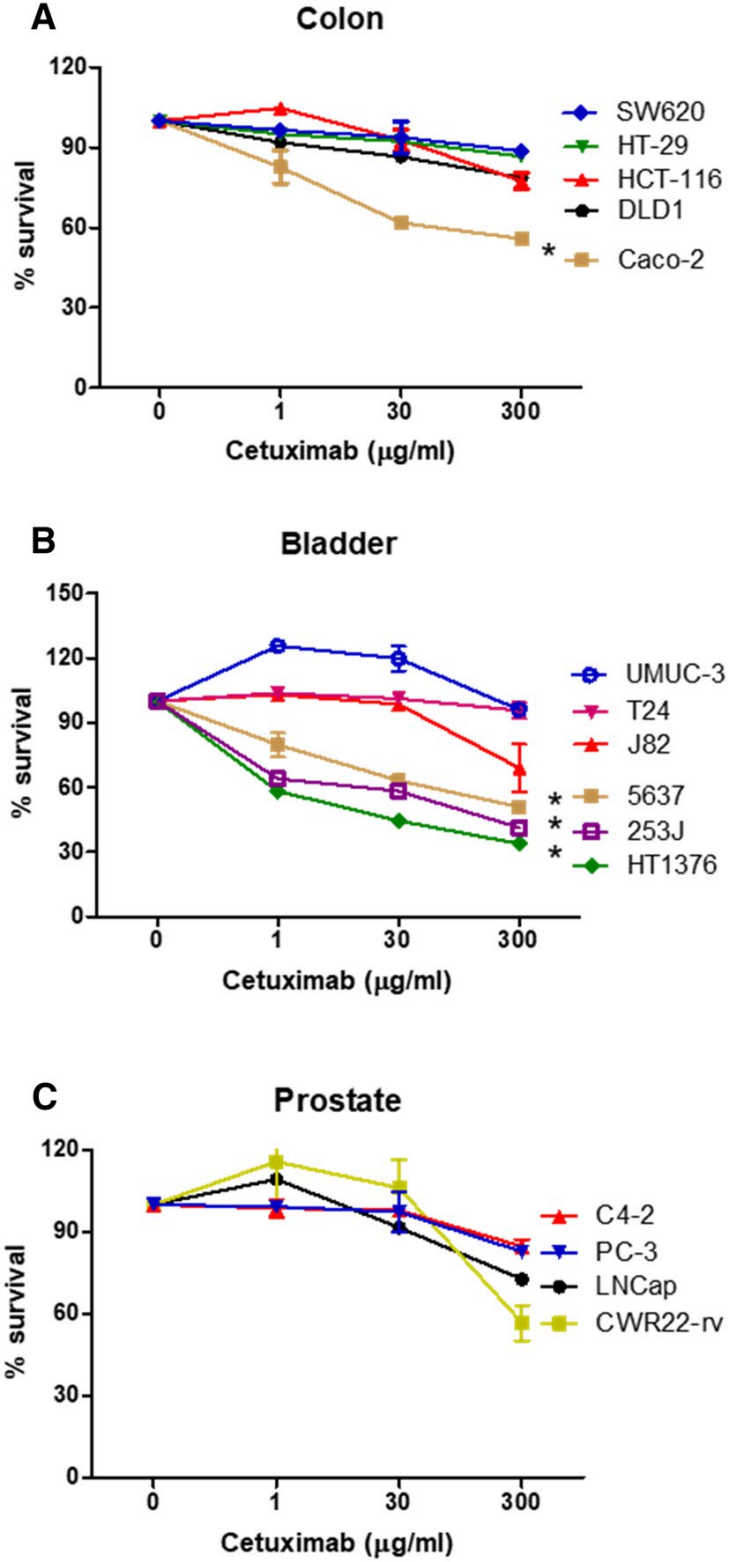
Effects of cetuximab on the colon, bladder, and prostate cancer cells. Cetuximab-mediated cancer cell killing was performed using the MTT cell proliferation assay. Cancer cells from colon (**A**), bladder (**B**), or prostate (**C**) were treated with cetuximab at concentrations ranging from 0 to 300 μg/ml for 72 h. The cells were stained with the MTT reagent, and the absorbance was measured at 570 nm. Experiments were repeated three times. The * in the diagram, determined with Turkey’s studentized range test, corresponds to statistically significant subgrouping of two groups (*p* < 0.05). Uppercase wt or m denotes retention of wild type or mutation of corresponding genes: SW620 (KRAS^m^), HT-29 (BRAF^m^/PIK3CA^m^), HCT-116 (KRAS^m^/PIK3CA^m^), DLD1 (KRAS^m^/PIK3CA^m^), Caco-2 (KRAS^wt^/BRAF^wt^/PIK3CA^wt^), UMUC-3 (KRAS^m^), T24 (HRAS^m^), J82 (PIK3CA^m^), 253J (PIK3CA^m^), 5637/HT1376 (not known mutations in EGFR signaling), CWR22-rv (PIK3CA^m^).

The EGFR-targeted bladder cancer therapies have been reported to yield mixed results with regards to their cancer-killing efficacy. In the current study, cetuximab-mediated cancer cell killing was investigated via in vitro MTT cell proliferation analysis of the colon, bladder, and prostate cell lines. Cancer cells were treated with cetuximab at concentrations ranging from 0 to 300 μg/ml, for up to 90 h. Data only showed cetuximab effects at 72 h. Most colon cancer cells used in this study were not sensitive to cetuximab owing to the mutations in RAS (DLD1, HCT-116, SW620), BRAF (HT-29), or the phosphatidylinositol-4,5-bisphosphate 3-kinase catalytic subunit alpha (PIK3CA) (DLD1, HCT116, HT-29)^31^ (Fig. 1A). Cetuximab effectively killed only Caco-2 cells (p < 0.05), which do not possess any mutations in the *KRAS, NRAS, BRAF,* and *PIK3CA* genes (Fig. 1A). Caco-2 cells were sub-grouped to the group of SW620, HCT-116, FT-29, and DLD1 (Turkey’s studentized range test, p < 0.05). Among the bladder cancer cell lines, HT1376, 5637, and 253J cells responded to cetuximab-mediated cytotoxicity in a dose-responsive manner (Turkey’s studentized range test, p < 0.05), whereas the J82, UMUC-3, and T24 cells were not killed by cetuximab (Fig. 1B). All cells, except UMUC-3, are known to retain the wild-type KRAS. While HT-1376 and 5637 cells have not been reported to retain any mutations in the EGFR signaling pathway, the 253J cells possess a mutation in the *PIK3CA* gene^31^. The T24 cells possess G12V mutations in H-RAS^32^. UMUC-3 contains the G12C K-RAS mutation, and J82 retains the PIK3CA mutation^31^. None of the tested prostate cancer cell lines responded well to cetuximab-mediated cell killing (Fig. 1C). *RAS* gene mutations are infrequent in prostate cancers. These data suggested that several bladder cancer cell lines with an intact EGFR pathway were as effectively killed by cetuximab as the colorectal cancer cells.

### Cetuximab effectively blocks EGFR signaling in the bladder cancer cells

**Figure 2:**
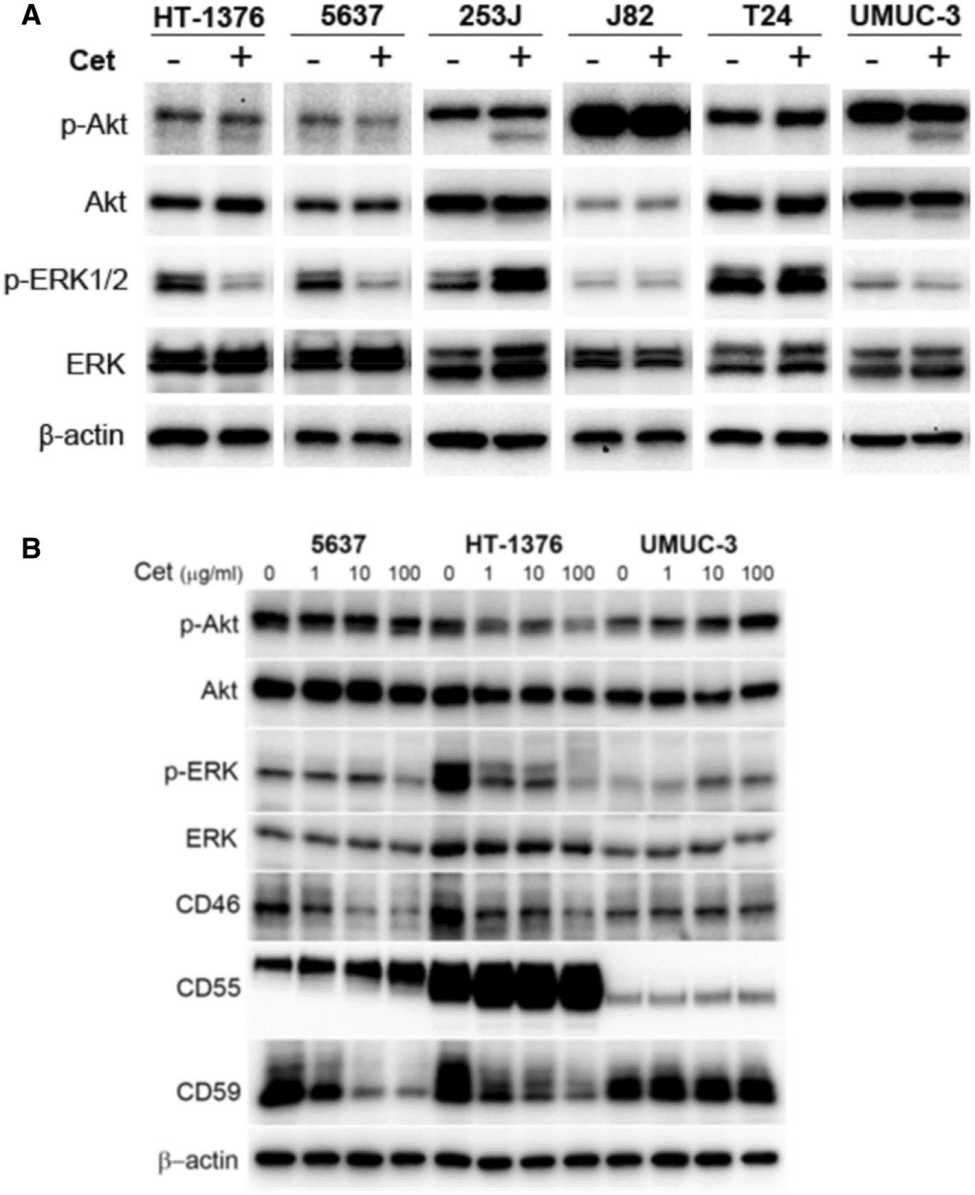
Effects of cetuximab on EGFR-dependent intracellular signaling in human bladder cancer cell lines. Corresponding cells were treated with cetuximab at a final concentration of 100 μg/ml (**A**) or 1–100 μg/ml (**B**). After 36 h, total cell protein extracts were subjected to immunoblotting with the indicated antibodies, as described in “Materials and methods”. Experiments were repeated three times with similar results.

To determine whether cetuximab inhibits the EGFR-mediated downstream signaling of AKT and ERK in the bladder cancer cells, these cells were treated with cetuximab at a final concentration of 100 μg/ml for 36 h. Subsequent western blot analysis revealed that the phosphorylated forms of AKT and ERK1/2 were downregulated by cetuximab in HT1376 and 5637 cells (Fig. 2A). Cetuximab more significantly downregulated expression of p-ERK in both cells. On the contrary, inhibition of AKT and ERK1/2 phosphorylation was not observed in 253J, J82, T24, and UM-UC-3 cells. It is noteworthy that HT1376, 5637, and 253J cells exhibited cetuximab-responsive cytotoxicity in Fig. 1. Next, to demonstrate the effect of cetuximab on the expression of mCRPs, including CD46, CD55, and CD59, HT1376 and 5637 cells were treated with 0–100 μg/ml cetuximab and subjected to western blot analysis. Cetuximab suppressed expression of CD46 and CD59 but stimulated expression of CD55 in both cells (Fig. 2B). Cetuximab also suppressed expression of p-AKT and p-ERK in HT1376 and 5637. In cetuximab-unresponsive UM-UC-3 cells, the expression of all mCRPs was not altered. These results suggested that both the HT-1376 and 5637 bladder cancer cells responded to cetuximab-mediated cell killing via the inhibition of AKT and ERK phosphorylation, and that mCRPs were involved in this process (Supplementary Information 1).

### CD46 restores cetuximab-mediated inhibition of AKT and ERK phosphorylation

**Figure 3:**
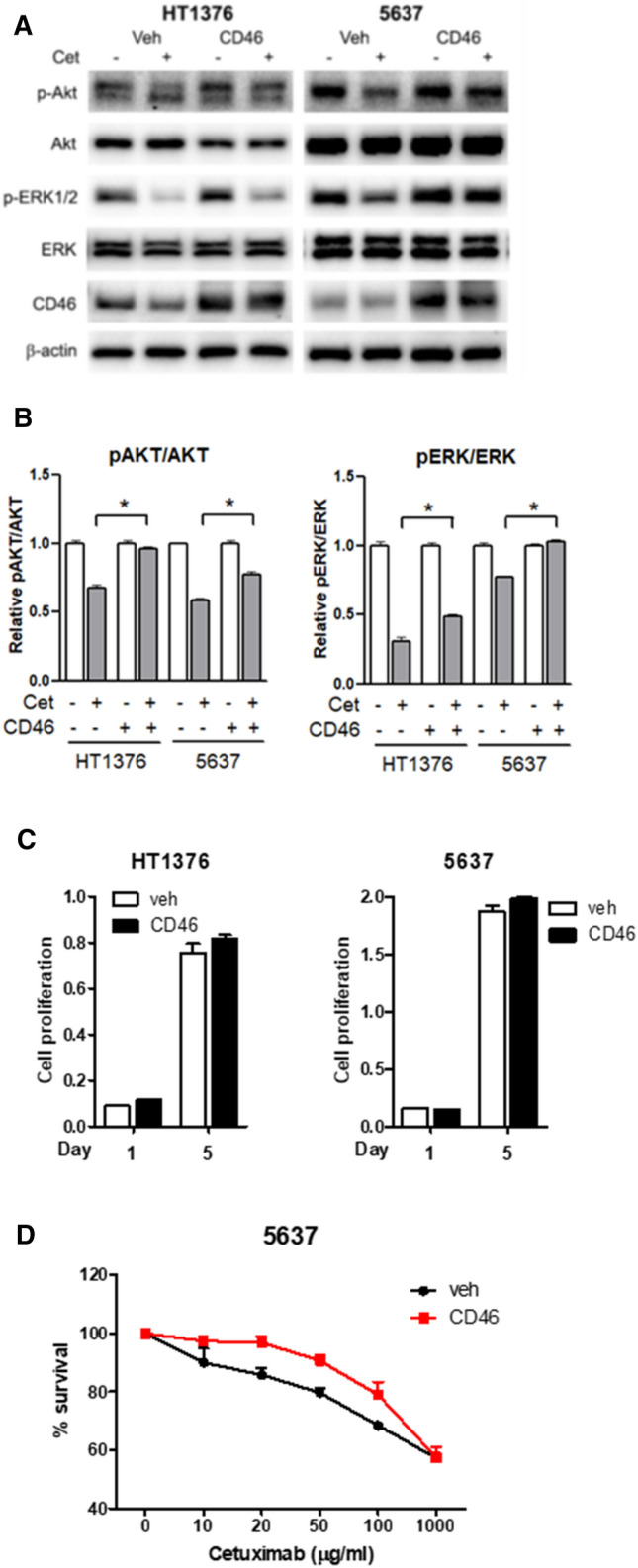
Restoration of the cetuximab-mediated inhibition of AKT and ERK phosphorylation by CD46. CD46 was overexpressed in HT-1376 and 5637 cells. The vehicle-loaded cells and the CD46-overexpressed cells were treated with 100 μg/ml cetuximab, incubated for 72 h, and analyzed via western blotting (**A**). Densitometric representation of relative expression of either pAKT or pERK was also shown (**B**). The MTT in vitro proliferation assay demonstrated cell growth of CD46-overexpressed cells in the absence (**C**) or presence of cetuximab (**D**). Cells were treated with cetuximab at concentrations ranging from 0 to 1,000 μg/ml for 72 h. The cells were stained with the MTT reagent, and the absorbance was measured at 570 nm. Experiments were repeated three times, and the density of each band was measured using a densitometer. Relative density of CD46 and p-AKT is shown as bar graphs. *p < 0.05 (two-tailed student t-test).

To investigate whether CD46 affects the downstream signaling of EGFR, CD46 was overexpressed, using lentivirus, in HT1376 and 5637 cells. Overexpression of CD46 restored both p-AKT and p-ERK1/2 expression that was suppressed by 100 μg/ml of cetuximab in HT1376 and 5637 cells (Fig. 3A). Expression of p-AKT and p-ERK was measured from three individual experiments, demonstrating that forced expression of CD46 restored cetuximab-mediated suppression of p-AKT and p-ERK at least in part in both HT1376 and 5637 cells (two-tailed student t-test, p < 0.05) (Fig. 3B). Restoration of p-AKT by CD46 was more obvious in HT1376 cells. To clarify that CD46 can affect bladder cancer cell proliferation without involvement of complement and/or lymphocytes, MTT in vitro proliferation assay was first performed in HT1376 and 5637 cells (Fig. 3C). The overexpression of CD46 did not significantly affect the proliferation of HT1376 and 5637 cells (p > 0.05). However, CD46 protected 5637 cells from cetuximab-mediated cell growth inhibition (p < 0.05; Fig. 3D). These results suggest that indirect effect of cetuximab-mediated growth inhibition of bladder cancer cells is protected by CD46 via regulation of p-AKT and p-ERK.

### CD46 protects the bladder cancer cells from both complement-dependent and antibody-dependent cell cytotoxicity

**Figure 4:**
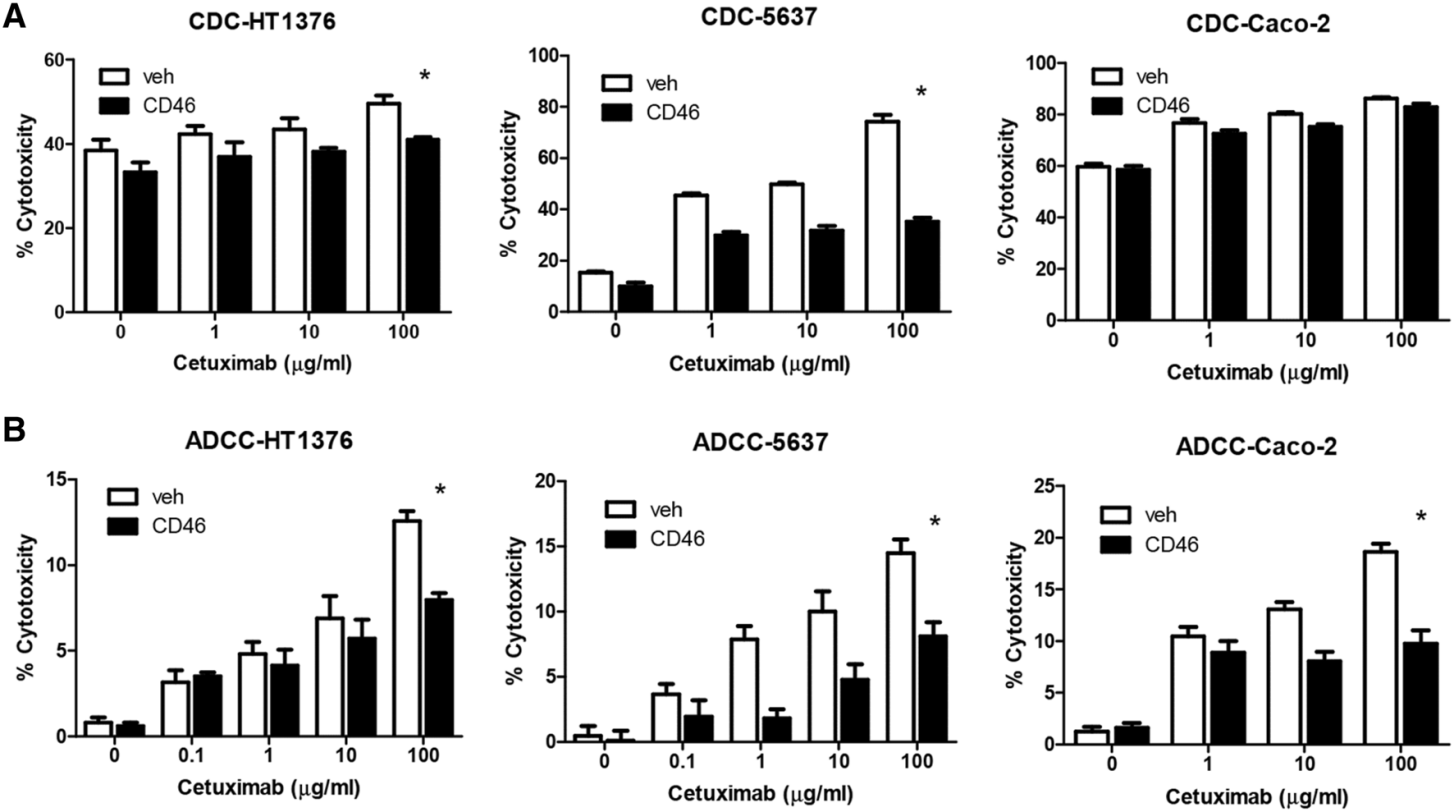
Protection of the bladder cancer cells from cetuximab-mediated cytotoxicity through both ADCC and CDC by CD46. Using HT1376, 5637, and Caco-2 cells, effects of CD46 on cetuximab-mediated CDC (**A**) and ADCC (**B**) were determined as described in “Materials and methods”. Caco-2 colon cancer cells were used as the reference cells. Experiments were repeated three times and results are expressed as mean ± standard deviation. *p < 0.05 (two-tailed student t-test).

Similarly to CD46, the primary function of mCRPs is to protect the cells from complement-mediated cell lysis^11^. While mAb therapies are used as targeted treatments against certain cancer cells, the role of complement and the associated proteins in modulating the antitumor effects of mAbs is still unclear^9,10,33^. In this study, the role of CD46 in protecting the cancer cells from CDC and cetuximab-mediated cell cytotoxicity was investigated. It has been previously reported by our group that CD46 is highly overexpressed in bladder cancer^13,34^. The MTT in vitro proliferation assay was first performed to determine whether CD46 overexpression can modulate the growth of cancer cells through modulating immunologic response. CDC assay revealed that the overexpression of CD46 protected both HT1376 and 5637 cells from complement-mediated lysis (p < 0.05; Fig. 4A). However, protective role of CD46 in Caco-2 was minimal against complement-mediated lysis (p > 0.05). Furthermore, CD46 protected the cells from cetuximab-mediated ADCC (p < 0.05; Fig. 4B). In Caco-2 cells, protective role of CD46 against ADCC was significantly enhanced (p < 0.05). These results suggested that CD46 protected the bladder cancer cells from both CDC and ADCC.

## Discussion

**Figure 5:**
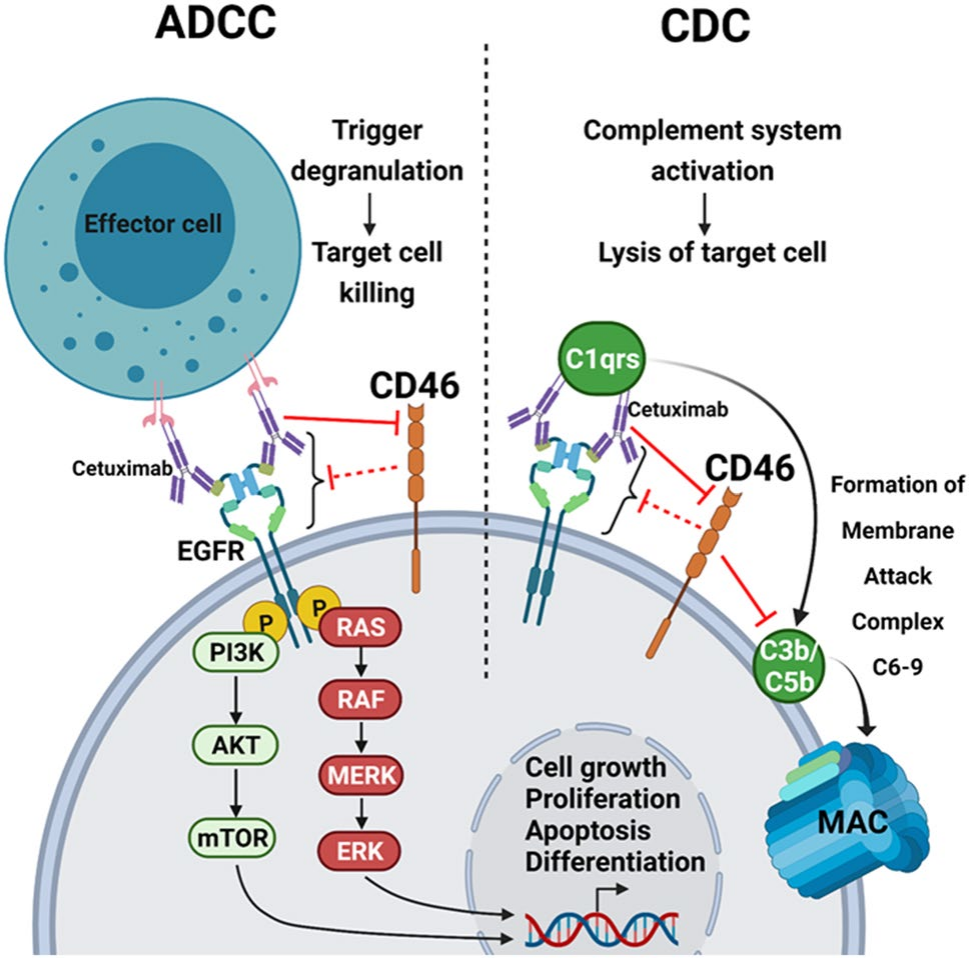
Schematic model of action mechanism of CD46 in the process of ADCC and CDC. ADCC occurs when the extracellular domain of the EGFR of target cell membrane is bound by the Fc region of cetuximab, blocking ligand binding, receptor dimerization, and intracellular signaling; this results in the lysis of the target cells via the production of cytokines and release of cytotoxic compounds. Natural killer (NK) cells are the typical ADCC effector cells. The complement system is also involved in modulating the anti-tumor activity of cetuximab. The Fc portion of cetuximab activates the complement C1 complex subsequently to form C3b and C5b deposited on the target cell surface, which leads to the formation of terminal complex called the MAC to lyse the cells. CD46 protects the host cells from CDC by binding to C3b, inactivating C3b and C4b, and preventing subsequent degradation by the plasma serine protease. Cetuximab ultimately induces cell death via both CDC and ADCC mechanisms. In this model, we propose that the cytotoxic effect of cetuximab is mediated by both ADCC and CDC mechanisms as well as via the downregulation of CD46 expression. Solid red lines denote CD46 downregulation by cetuximab as demonstrated in this study. Dotted red lines suggest unknown mechanism of protective role of cetuximab mediated indirect effects as well as direct effects through the regulation of ADCC and CDC. “Created with BioRender.com”.

Mechanisms contributing to mAb-induced tumor cell death include direct and indirect effects^35^. Direct effects are exerted through cross-linking of receptors or via blockade of receptor-ligand binding, such as in CDC and ADCC. Indirect effects include modulation of the tumor microenvironment. Complement activity affects the CDC as well as the action of mAbs with both additive and antagonistic effects^36–38^. ADCC is the primary action mechanism of various anticancer mAbs^33^. ADCC occurs when antigens of the target cell membrane are bound by the Fc region of the mAbs (Fig. 5). This results in target cell lysis via the production of cytokines and the release of cytotoxic compounds. Natural killer (NK) cells are the typical ADCC effector cells. The complement also plays an important role in modulating the antitumor activity of various mAbs. The Fc portion of mAbs activates the complement component C1 that cleaves C4 and C2 to form the C3 convertase, which further cleaves C3 into C3a and C3b^39^. C3b participates in the formation of C5 convertase that cleaves C5 into C5a and C5b. C3b and C5b are deposited on the target cell surface to form the subsequent terminal complex called the MAC that lyses the cells. Several reports support CDC as an effective target mechanism of mAbs. For example, complement activation is important for the action of rituximab, the mAb targeted against CD20 in the lymphomas^37,40^. Additionally, CDC induction plays important roles in alemtuzumab (CD52) therapy for leukemia and the combined therapy with trastuzumab and pertuzumab (anti-HER2/neu) for breast and gastric cancers^41–43^. Another anti-CD20 mAb of atumumab enhances the CDC-mediated cell killing under both in vitro and in vivo conditions^44,45^. Nevertheless, the efficiency of CDC-mediated tumor cell killing under in vivo conditions is somewhat debatable, partly due to the overexpression of mCRPs in tumors^21,46^. These mCRPs limit MAC formation and cancer cell lysis.

The mCRPs inhibit complement activation in various cancers^47,48^. Among them, CD46, CD55, and CD59 prevent MAC formation and lysis of the host cells. CD46 protects the host cells from CDC by binding to C3b, inactivating C3b and C4b, and preventing their subsequent degradation by the plasma serine protease^12^. CD55 prevents CDC by accelerating the decay of C3, and C5 convertases and CD59 inhibit the formation of MAC, thereby preventing complement-mediated lysis^11^. Of all mCRPs, CD59 is considered to be the most effective and acts additively; in certain tumors, it acts synergistically with CD55 and CD46^49^. Silencing of CD46 causes a remarkable increase of 20–30% in the CDC. The increment in the CDC by the silencing of CD55 and CD59 was found to be 24% and 55%, respectively^50^. The mCRPs are highly expressed in malignant cells^51^. For example, CD46 is usually overexpressed in tumors such as hepatocellular carcinoma, multiple myelomas, and bladder and colon cancers^34,49,52–54^. The mCRPs add another layer of complexity: they are involved in mAb-induced CDC as well as in ADCC^33^. Considering that all mCRPs can directly block ADCC in the target cells^19–22^, overexpression of CD46 could be a survival pathway of the bladder cancer cells to avoid innate immunity. There is a considerable amount of evidence supporting the role of mCRPs as the protectors of mAb-induced CDC and ADCC. Silencing of CD46, CD55, or CD59 enhanced ADCC, even in the absence of the complement^20,22,55^, suggesting that mCRPs inhibit ADCC as well as CDC. In this study, cetuximab inhibited the expression of CD46 and CD59, but stimulated that of CD55, thereby protecting cancer cells from both CDC and ADCC. This study was limited to elucidating the role of CD46 in the regulation of CDC and ADCC in bladder cancer cells. Overexpression of CD46 protected the cancer cells from both CDC and ADCC, suggesting that the EGFR-targeted anticancer agent cetuximab downregulated the expression of CD46 to maximize the cell-killing activity, thereby affecting both CDC and ADCC. While the mechanism of the regulation of mCRP expression was not investigated, cytokine release from NK cells activated by mAbs could be involved in the alteration of mCRP expression. In fact, Blok et al. demonstrated that the expression of CD46 and CD59 is downregulated by interleukin-1β, the expression of CD46 is downregulated by IL-4, and that of CD46 and CD55 is downregulated by TGF-β1 in renal cancer cells^56^.

In summary, we demonstrated that the EGFR inhibitor cetuximab could be an effective targeted therapeutic agent for bladder cancers that require better treatment methodologies. Cetuximab enhanced the therapeutic effect in bladder cancer cells by affecting the EGFR signals via both ADCC and CDC-based mechanisms. While treatment of cancer cells with cetuximab caused the downregulation of CD46 and CD59, the overexpression of CD46 protected the cells not only from direct effects via ADCC and CDC but also through indirect effects (Fig. 5). The results presented here indicated that the cetuximab-mediated inhibition of CD46 expression might be a beneficial action mechanism in mAb immunotherapy for cancers.

## Materials and methods

### Cell lines and cell culture

Human bladder cancer cell lines (T24, J82, 5637, HT1376, UMUC-3, and 253J), prostate cancer cell lines (LNCaP, CWR22rv, C4-2, and PC-3), and colon cancer cell lines (DLD1, Caco-2, HCT116, HT-29, and SW620) were purchased from the American Type Culture Collection (ATCC, Manassas, VA, USA). Colon cancer cell lines were cultured in Dulbecco's modified Eagle's medium (DMEM, Welgene, Korea). The other cell lines were maintained in the Roswell Park Memorial Institute-1640 medium (RPMI, Welgene, Korea). The complete media were supplemented with 5% heat-inactivated fetal bovine serum (FBS, Gibco, Life Technologies, Grand Island, NY, USA) and 1% penicillin/streptomycin (Gibco) before use. All cultures were maintained at 37 °C and 5% CO_2_, and the medium was renewed every 3–4 days. Overexpressing CD46 cell lines were generated by using the lentiviral vector pBlasti-eGFP-CD46 as described previously^57^. CD46 overexpression clones were grown in a medium supplemented with 10 µg/ml blasticidin (Sigma-Aldrich, St. Louis, MO, USA).

### Western blotting

Cells were lysed in the RIPA buffer supplemented with cocktails of protease/phosphatase inhibitors (Cell Signaling Technology, Beverly, MA, USA). Proteins (20 μg) were separated using a 10% SDS–polyacrylamide gel and the Bio-Rad electroporation system and then transferred onto PVDF membranes (Millipore, Billerica, MA, USA). CD46 antibodies were obtained from OriGene Technologies, Inc. (TA306994, Rockville, MD, USA).

CD55 (sc-51733), CD59 (sc-133170), p-AKT (sc-514032), AKT (sc-5298), p-ERK1/2 (sc-136521), and ERK1/2 (sc-514302) were procured from Santa Cruz Biotechnology (SantaCruz, CA, USA). β-Actin antibodies were obtained from Sigma-Aldrich (A5441, St. Louis, MO, USA). The bands were visualized and analyzed using the Immobilon Western detection system (Millipore, Billerica, MA, USA) and ChemiDOC™ MP Gel Imaging System (Bio-Rad, Hercules, CA, USA).

### Growth inhibitory assay

The growth inhibitory effect of cetuximab in cell lines was analyzed using the MTT assay. For this, 5000 cells in 0.5 ml media were seeded in each well of a 24-well microtiter plate. The next day, these cells were treated with various concentrations of cetuximab (from 0 to 300 µg/ml) and cultured for three days. The anti-EGFR cetuximab (Erbitux 5 mg/ml, Merck Co., Ltd.) was provided by the Chonnam National University Hwasun Hospital. Cells were incubated in the MTT solution for 4 h. The crystals obtained were dissolved in DMSO, and the absorbance was measured at 570 nm using a microplate reader with SoftMax Pro software (Molecular Devices, Sunnyvale, CA, USA).

### Cytotoxicity assay

The assays to examine cetuximab-mediated ADCC and CDC were performed with the CytoTox 96 Non-Radioactive Cytotoxicity Assay Kit (Promega, Madison, WI, USA) according to the manufacturer’s protocol. For evaluation of the ADCC activity, whole blood was obtained from healthy volunteers after they provided informed consent to participate in this study at Chonnam National University Hospital. The research attained ethical approval from the institutional review board of Chonnam National University Hospital (IRB No. 06–070). Peripheral blood mononuclear cells (PBMC) were isolated via Ficoll–Paque density gradient centrifugation and used as the effector cells. The target cells were suspended in RPMI medium without FBS, at a concentration of 10^5^ cells/ml, and 0.1 ml of this culture was seeded in each well of the 96-well U-bottom microtiter plates. After 24 h, the cells were exposed to various concentrations of cetuximab (0–100 μg/ml) and incubated for 1 h. Next, the effector cells were added at an effector to target cell ratio of 10:1. For evaluating the CDC activity, human serum from a healthy volunteer was used as the source of the complement. To yield a 1:3 final dilution, 50 μl serum was added. All experiments were performed in triplicate in individual wells. The plates were incubated for 4 h at 37 °C, and the absorbance of the supernatants was recorded at 490 nm to determine the release of lactate dehydrogenase. Calculations for assessing the ADCC and CDC were performed according to the following formula:

$$\mathrm{For\,ADCC:}\mathrm{ \%cytotoxicity }= (\mathrm{experimental\,release }-\mathrm{effector\,spontaneous\,release }-\mathrm{ target\,spontaneous\,release})/ (\mathrm{target\,maximum\,release }-\mathrm{target\,spontaneous\,release}) \times 100.$$

$$\mathrm{For\,CDC:}\mathrm{ \% cytotoxicity }= (\mathrm{experimental\,release }-\mathrm{spontaneous\,release}) / (\mathrm{maximum\,release }-\mathrm{spontaneous\,release}) \times 100.$$

### Statistical analysis

The SPSS 21.0 software (IBM, Chicago, IL, USA) was used for statistical analysis; and all data were presented as mean ± standard deviation. Data were analyzed using either one-way or two-way ANOVA. Cell growth assays were analyzed by Turkey’s studentized range test for yield. The p values less than 0.05 were considered statistical significant.

### Ethical approval

All procedures performed in studies involving human participants were in accordance with the ethical standards of the institutional research committee and with the 1964 Helsinki declaration and its later amendments or comparable ethical standards. Written informed consent was received from the patients involved.

## Supplementary Information

Supplementary Information.

## Data Availability

The datasets used and/or analyzed during the current study are available from the corresponding author on reasonable request.
