# Supplementary material for: CD46 protects the bladder cancer cells from cetuximab-mediated cytotoxicity
Source: Sci Rep. 2022 Dec 27;12:22420. doi: 10.1038/s41598-022-27107-9 (PMC9794803; doi:10.1038/s41598-022-27107-9)
Supplement: Supplementary file 1 — Supplementary Information. [file 41598_2022_27107_MOESM1_ESM.pdf]

Figure 2A – HT1376, J82 cells

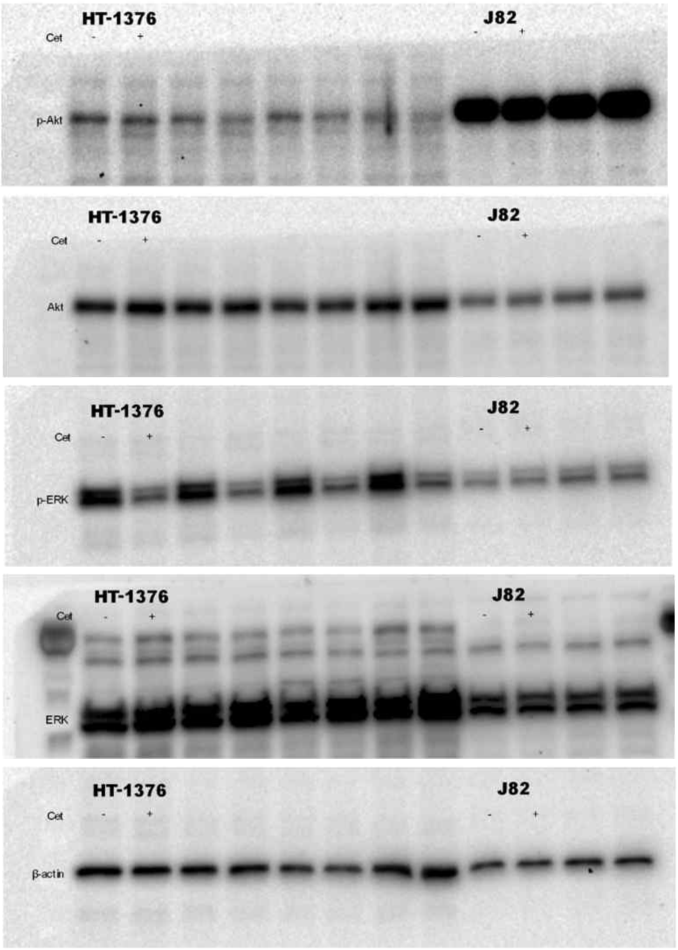

Figure 2A – HT1376, J82 cells

Less exposure of page 1

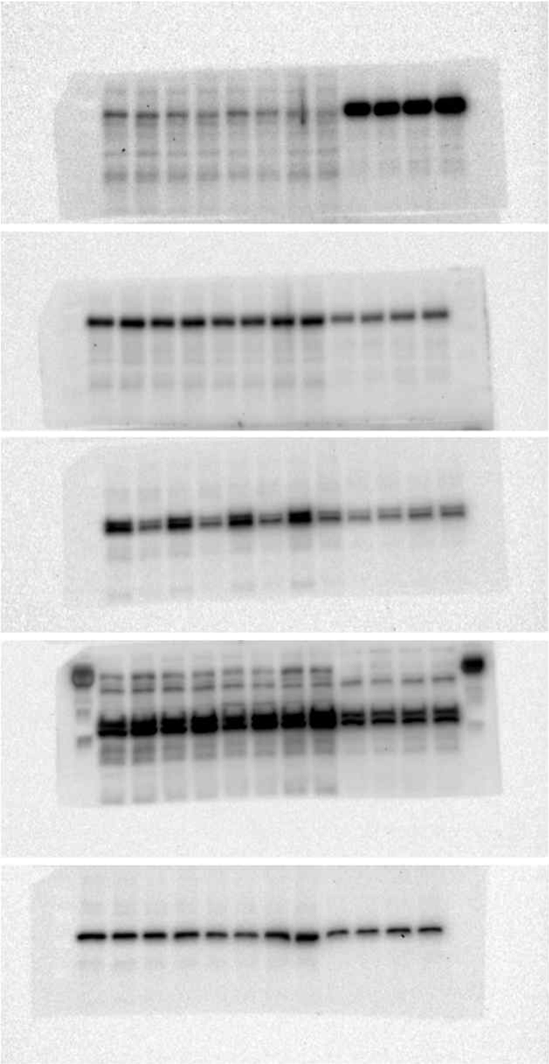

Figure 2A – 5637 cells

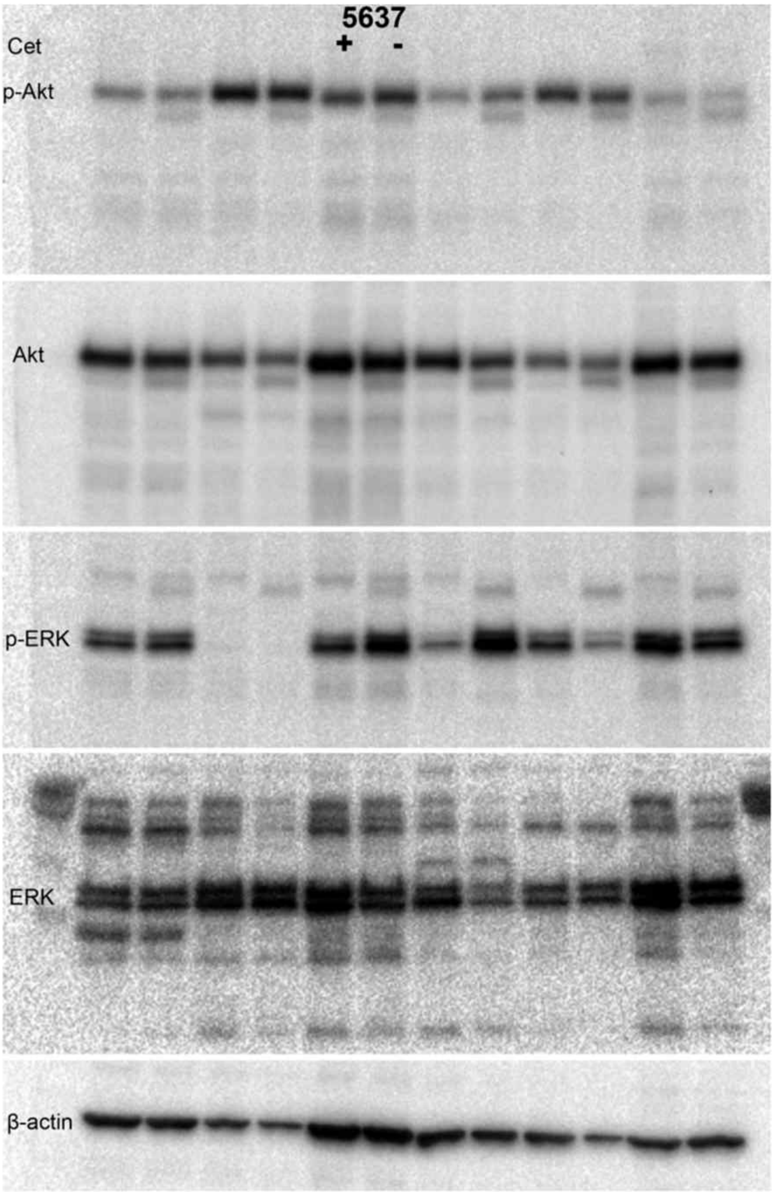

Figure 2A – 5637 cells

Less exposure of page 3

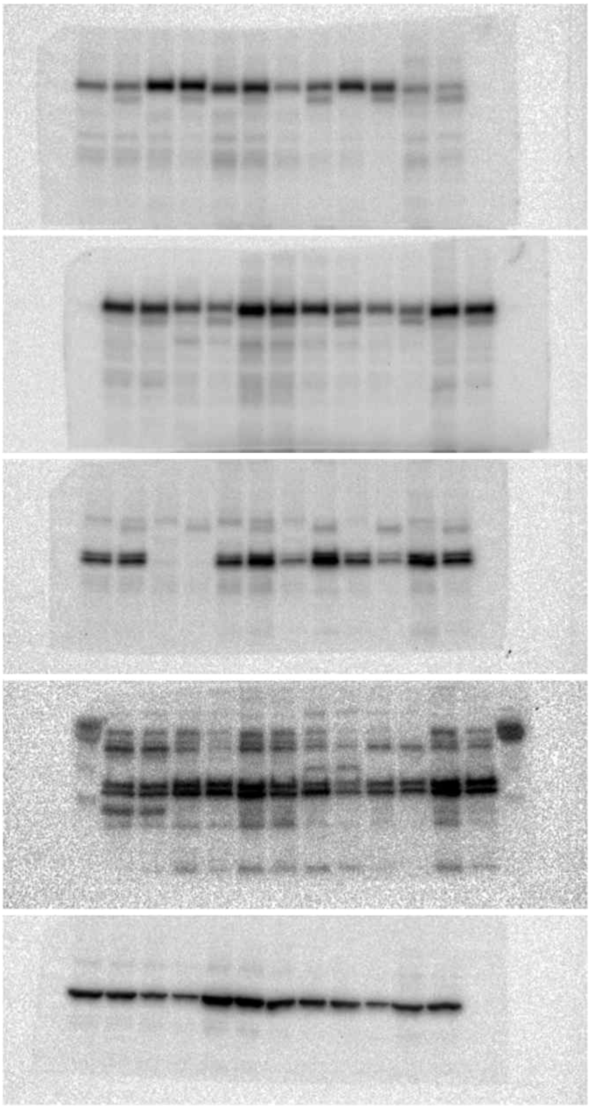

Figure 2A – T24 cells

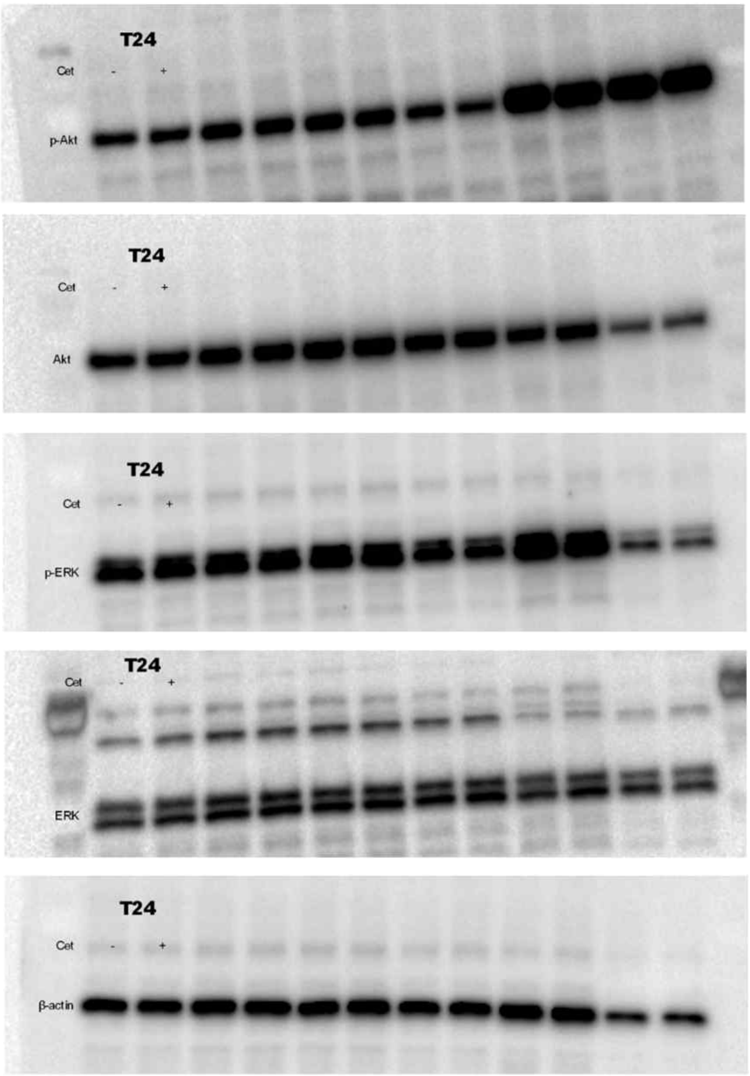

Figure 2A – T24 cells

Less exposure of page 5

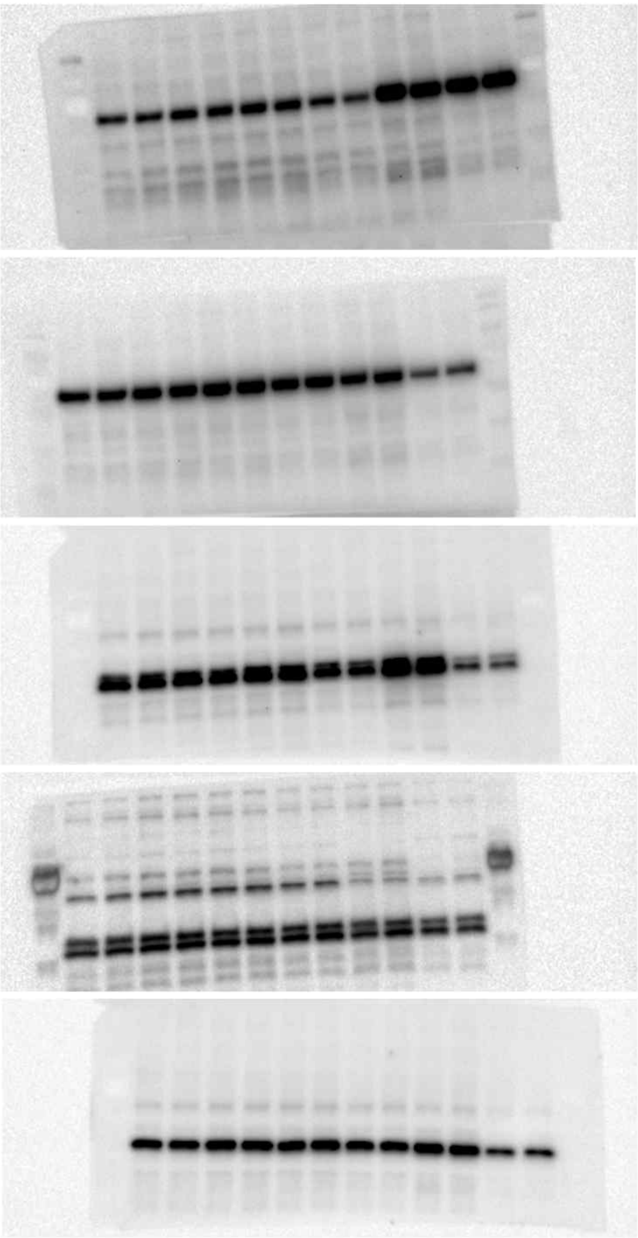

Figure 2A – UMUC-3, 253J cells

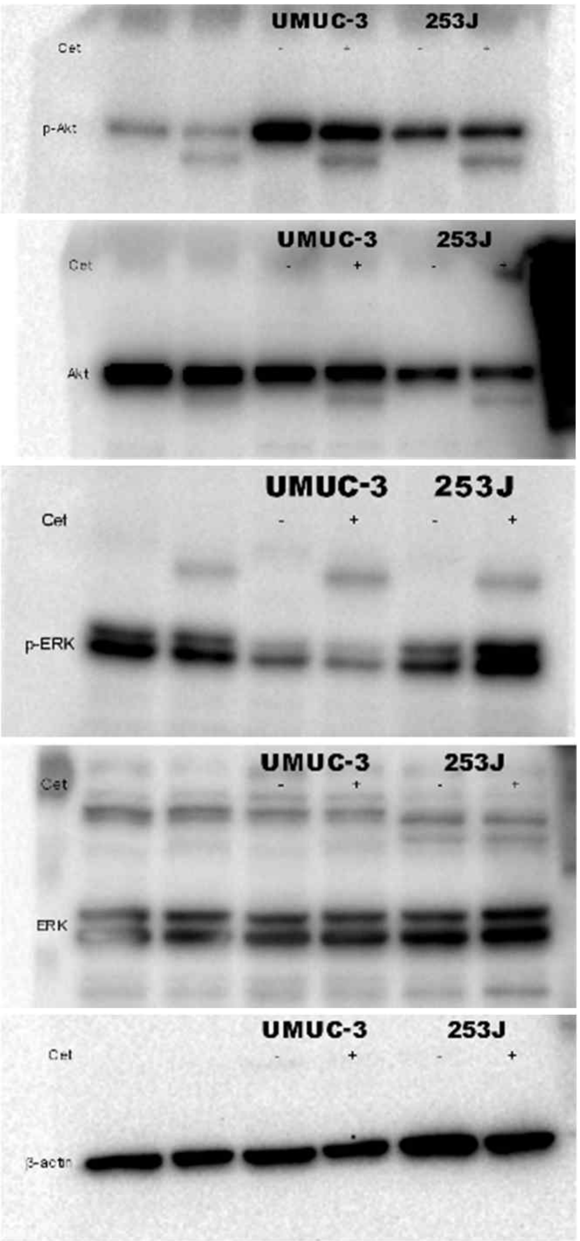

Figure 2A – UMUC-3, 253J cells

Less exposure of page 7

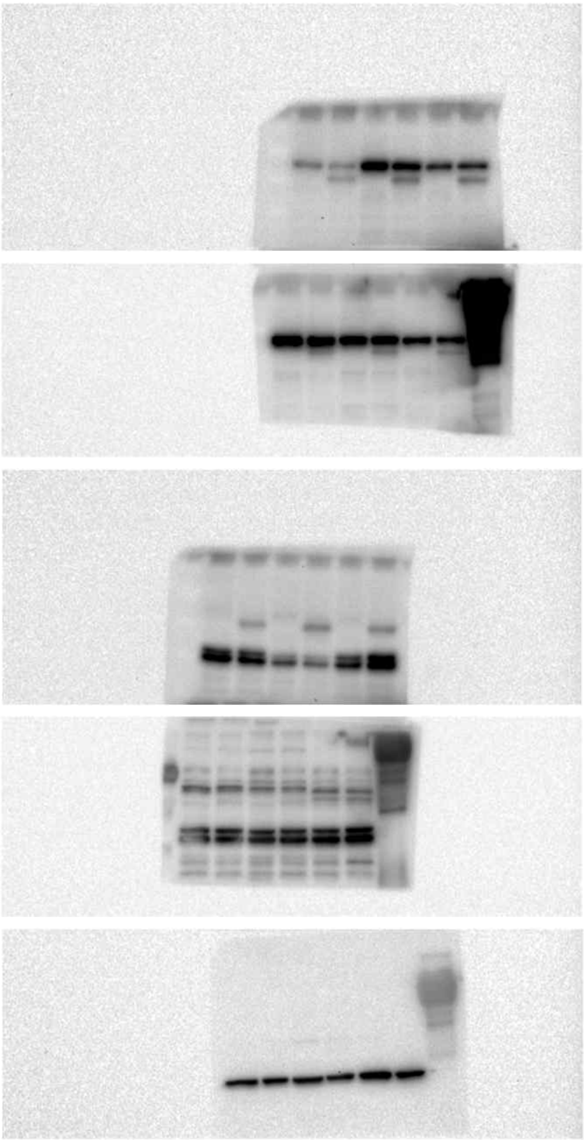

Figure 2B

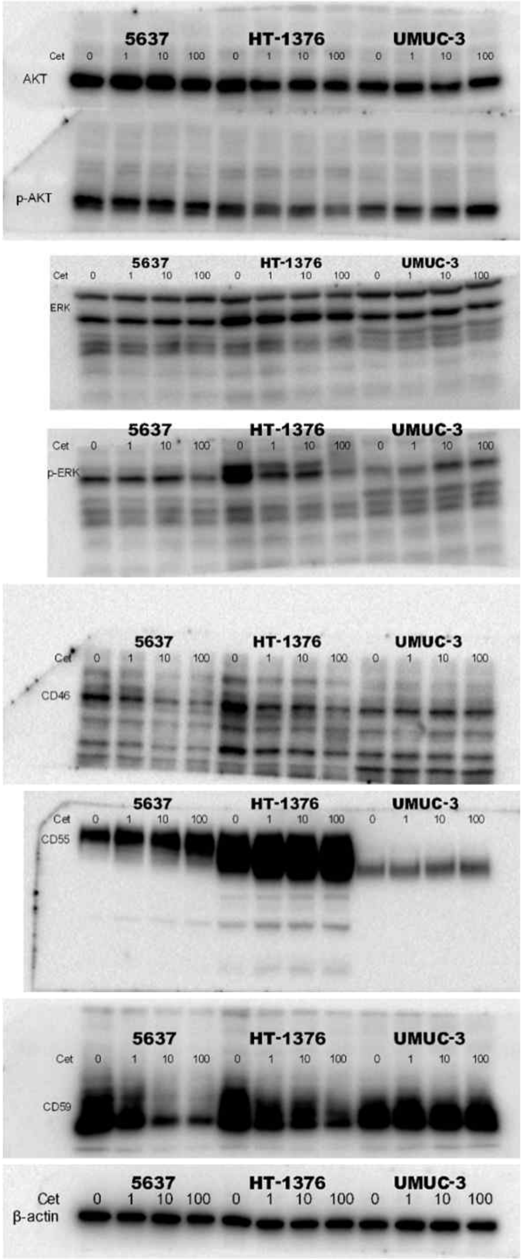
5637
HT-1376
UMUC-3
Cet
0
1
10
100
AKT
p-AKT
ERK
p-ERK
CD46
CD55
CD59
β-actin

Figure 2B – AKT, pAKT, ERK, pERK

Less exposure of page 9

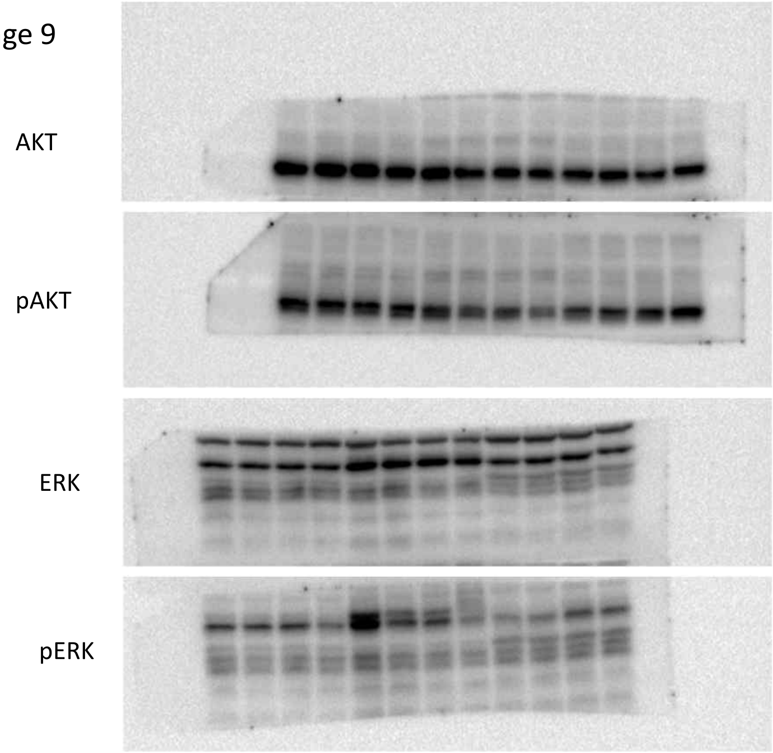

Figure 2B – CD46, CD55, CD59

Less exposure of page 9

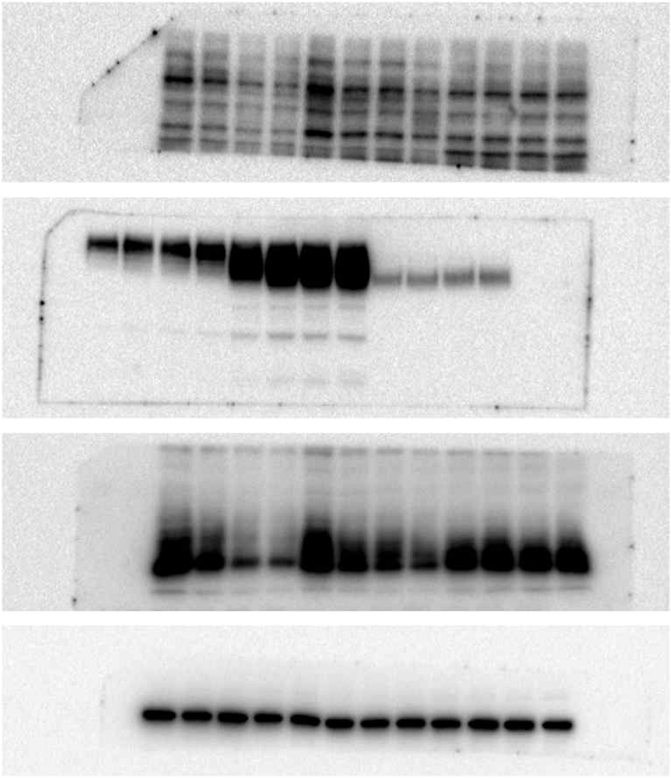

Figure 3A

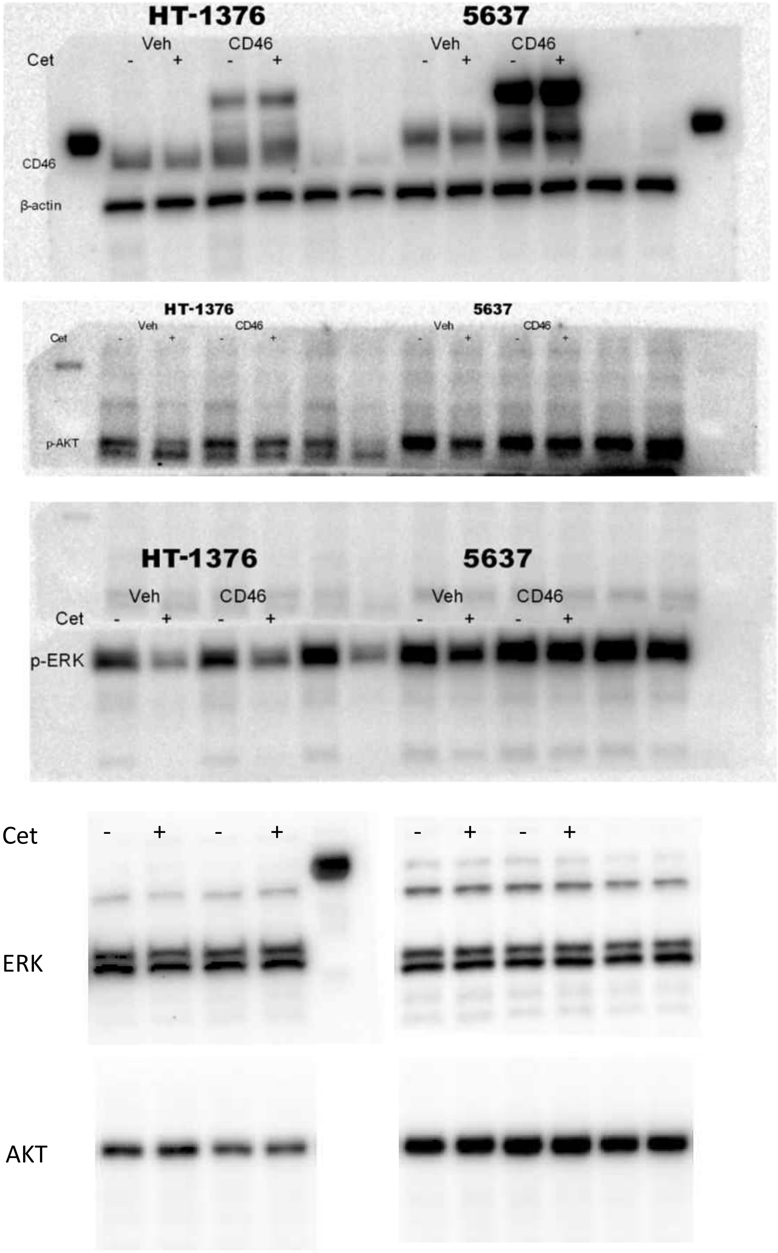
HT-1376
5637
Veh
CD46
Cet
- + - +
- + - +
CD46
β-actin
HT-1376
5637
Veh
CD46
Cet
p-AKT
HT-1376
5637
Veh
CD46
Cet
- + - +
- + - +
p-ERK
Cet
- + - +
- + - +
ERK
AKT
